# An estimation of the effect of finding nurses helpful on early breastfeeding exclusivity mediated via breastfeeding self-efficacy, using a counterfactual approach and G-computation

**DOI:** 10.1186/s12884-025-08429-8

**Published:** 2025-12-30

**Authors:** Alessandra Prioreschi, Shane Anthony Norris, Cindy-Lee Dennis

**Affiliations:** 1 SA MRC/Wits Developmental Pathways for Health Research Unit, Department of Paediatrics, Faculty of Health Sciences, School of Clinical Medicine, University of the Witwatersrand, Johannesburg, South Africa; 2 School of Health and Human Development, University of Southampton, Southampton, UK; 3 Lawrence S. Bloomberg Faculty of Nursing, University of Toronto, Toronto, Canada; 4 Lunenfeld-Tannenbaum Research Institute, Sinai Health, Toronto, Canada; 5 Department of Psychiatry, Faculty of Medicine, University of Toronto, Toronto, Canada

**Keywords:** Breastfeeding support, Early cessation, Exclusivity, Policy recommendations

## Abstract

**Background:**

Exclusive breastfeeding is recommended for at least the first six months of life, however in South Africa only 32% of mothers are exclusively breastfeeding to six months and discontinuation happens early. Nurses are a key resource for promoting breastfeeding self-efficacy and thus exclusivity, yet mothers often report nurses to have poor attitudes, which deter health seeking behaviour. This study aimed to infer whether perceived support from nurses immediately postpartum was causally related to breastfeeding exclusivity, and specifically whether this effect was mediated via breastfeeding self-efficacy.

**Methods:**

This cross-sectional study recruited mothers from two community clinics in Soweto and collected data on breastfeeding practices, breastfeeding self-efficacy, and mothers’ perceived support received from nurses within the first few days following delivery. To estimate the causal effect, G-computation for mediation analysis with Monte Carlo simulation was used. In all cases, 1000 bootstrapped samples were created to tighten the estimates, and bias corrected 95% confidence intervals were presented.

**Results:**

Data from 169 participants were included in the analysis. Most participants (55%) scored 70/70 on the breastfeeding self-efficacy scale, and only 14% scored below 56/70 (lowest quartile). Less than half of the women found the nurses helpful ‘always’. Most data were collected within 4 days of birth (IQR: 3–7 days), with only 10% being collected after 12 days. While nearly 90% of women were breastfeeding at this time, only 78% were exclusively breastfeeding. The results from the mediation analysis show that there was a total causal effect of finding nurses helpful on breastfeeding exclusivity, whereby finding nurses ‘always’ helpful resulted in a 14% greater likelihood of exclusively breastfeeding (SE = 0.06, *p* = 0.03, BC 95% = 0.00 to 0.24). However, this effect was not mediated via breastfeeding self-efficacy (proportion mediated = 7%; NIE = 0.01, BC 95%: -0.00 to 0.05).

**Conclusions:**

There is evidently a need for proper training for healthcare workers in supporting mothers and providing antenatal education and anticipatory guidance around breastfeeding given the impactful role they have on breastfeeding decision making. We recommend that interventions to promote breastfeeding exclusivity need to happen prior to delivery, or immediately upon delivery. Furthermore, all clinics need to enforce the BFHI immediately to provide mothers with a chance at exclusivity.

**Supplementary Information:**

The online version contains supplementary material available at 10.1186/s12884-025-08429-8.

## Background

Breastfeeding provides the complete nutritional requirements for infants in the first six months of life, and with the introduction of solid foods continues to provide essential nutrition and immunological protection in the first two years of life or longer [1]. Exclusive breastfeeding for the first six months improves infant cognitive function, metabolic health and bonding between the mother-infant dyad [2, 3]. Furthermore, mothers who breastfeed may be provided some protection against cancers and type II diabetes [2, 3]. Despite these well-documented benefits, only 48% of children globally are exclusively breastfed for six months with the premature discontinuation of breastfeeding accounting for 16% of child mortality each year [2]. South Africa has one of the lowest rates globally with only 32% of mothers exclusively breastfeeding to 6 months and many discontinuing exclusivity by 2.9 months on average [4]. Recommendations from UNICEF and the WHO are that infants initiate breastfeeding within the first hour of birth, which is critical for newborn survival and establishing breastfeeding behaviours and milk supply [5].

There are various global policies to support breastfeeding exclusivity, such as The International Code of Marketing of Breast-Milk Substitutes, which restricts the promotion of any substitutes to breastfeeding; as well as local policies protecting maternity leave, and workplace breastfeeding policies to support working mothers. However, the implementation of these policies is variable. The Lancet Breastfeeding Series (2023) highlights that more children than ever before are being given breastmilk substitutes [6], and in South Africa both civil societies and medical professionals have taken action to protect these policies. However individual groups cannot stem the actions of massive corporations, and regulation is needed from ministries, professional associations, and education institutions. The Ten Step Baby-Friendly Hospital Initiative [7] (BFHI) encourages integration of ten practices to improve breastfeeding initiation and exclusivity at all maternity care facilities, yet globally only 14% of countries report the majority of births occurring at Baby-Friendly facilities [2]. While South Africa has adopted this policy [8, 9], it has not been systematically implemented and monitored for uptake and it is not included in strategic performance plans or budgeting [9]. Following the Tshwane Declaration of Support for Breastfeeding in South Africa in 2011, 75% of public hospitals were accredited as Baby-Friendly by 2015 [9]. While breastfeeding benefits have been reported in accredited facilities, specifically in initiation rates and the promotion of breastfeeding positive maternity practices such as skin-to-skin contact and feeding on demand, no improvements in exclusivity rates have been documented [9]. In South Africa a relatively high number of infants (67%) initiate breastfeeding within the first hour of life compared to the 46% rate reported globally [2]. It is interesting that there are such low rates of exclusivity at six months given the relatively high rates of early initiation, while globally, initiation and exclusivity rates are similar. It seems there is a crucial period immediately following initiation when intervention should be prioritised.

Proper breastfeeding support and education are known to increase breastfeeding self-efficacy and therefore exclusivity [10, 11]. Nurses are a key resource for promoting breastfeeding self-efficacy, in that they can provide verbal persuasion, improved performance accomplishments, and provide opportunities for vicarious experience. Nurses’ knowledge about breastfeeding and attitudes towards breastfeeding are predictive of their behaviour in supporting mothers, and maternal perceptions of negative or neutral attitudes from hospital staff towards breastfeeding have been shown to be predictive of breastfeeding failure at 6 weeks following delivery [12]. In developing countries health workers are seen to be in a position of power and this perceived hierarchy makes their influence on mothers’ decision making even higher [13]. However, in South Africa mothers frequently report that nurses are rude, judgmental and even abusive [14–16], which impacts health seeking behaviour.

Ultimately, it is essential to understand maternity care practices and factors impacting South African mothers that result in discontinuation of exclusive breastfeeding well before six months even when initiation happens early. Mothers in South Africa are often discharged before breastfeeding behaviours and milk supply have even been established [9], and Baby-Friendly Initiatives are often not implemented in community clinics where women seek breastfeeding support [9]. It is unknown exactly what causes discontinuation so early on in South African women, however it is likely that if mothers found healthcare workers approachable and helpful they would seek and receive more support for breastfeeding in the first few days following delivery, which may increase breastfeeding self-efficacy and thus exclusivity. Therefore, this study aimed to determine infant feeding practices shortly following delivery, and to use counterfactual approaches to infer whether perceiving clinic sisters (nurses) to be helpful in general was causally related to breastfeeding exclusivity in the first few days and weeks postpartum, and specifically whether this effect was mediated via breastfeeding self-efficacy. This research will assist in the development of preventive interventions to support mothers in breastfeeding exclusively to 6 months postpartum and beyond.

## Methods

This cross-sectional study is a sub-study of the PLAY trial (Play, Love, And You) which aimed to evaluate the effectiveness of a supportive intervention on maternal self-efficacy among mothers in South Africa from shortly after delivery until 12 months postpartum. Further details about the PLAY trial can be found in the protocol manuscript [17]. This sub-study consists of cross-sectional data collected when participants were recruited, prior to being randomized or initiating the intervention.

### Participants

Mothers and their newborns (*n* = 201) were recruited from two community clinics in Soweto, South Africa – Lillian Ngoyi Community Clinic, and Chiawelo Community Healthcare Centre. Women were eligible to participate if they (1) resided and intended to remain in Soweto for the duration of the PLAY trial (2) were >= 18 years old, (3) delivered a singleton infant at > 36 weeks gestational age, (4) had not previously been diagnosed with or were currently medicated for epilepsy, type 1 diabetes or tuberculosis (TB), and (5) were the primary caregiver for the infant. All women completed informed consent procedures approved by the Human Research Ethics Committee at the University of the Witwatersrand (M220217).

### Data collection

All data were initially collected at the community clinics, while mothers were waiting for infant immunisations, or during regular postpartum clinic checkups. Participants were interviewed by trained research assistants who entered data directly into the REDCap electronic data capture and management system hosted at the University of the Witwatersrand [18, 19]. For a sub-sample of participants, data collection was repeated at the participant’s home if they had not been able to provide complete data on all PLAY Study trial variables at the community clinic (for example, if they ran out of time), and this could have occurred on the same day, or many days later.

### Exposure: Perceived support from nurses

Mothers completed a 9-item self-report questionnaire assessing social support in the antenatal period, which has previously been used in this context [20, 21]. In this questionnaire, mothers were asked a question about their general perceptions of nurse support (i.e.: “Some people think the sisters at the clinic (nurses) are always helpful, others think only sometimes and some say they are seldom helpful. How do you feel?”), and this variable was collapsed into a binary variable: finding nurses helpful always (1) or finding nurses helpful sometimes, seldom or never (0).

### Outcome: Breastfeeding practices

Mothers were asked a series of infant feeding questions including whether they were currently breastfeeding (yes/no), and if not at what infant age (in days) they stopped breastfeeding and why. They were asked whether their infant was put to their breast within the first hour following delivery (yes vs no), whether they had ever given their newborn anything besides breastmilk (yes vs no), and if so, what they fed their newborn. Mothers reported if they had ever given their newborn formula (yes vs no), whether they were currently feeding formula (yes vs no), as well as how old the newborn was when it was first given anything other than breastmilk (including formula) in days. Using this information, it was determined whether the mother was exclusively breastfeeding (yes vs no). Mothers were considered to be exclusively breastfeeding if they were currently breastfeeding and had never given their newborn anything besides breastmilk. This questionnaire is included as Supplementary File 1.

### Mediator: Breastfeeding self-efficacy

Maternal breastfeeding self-efficacy was measured using the breastfeeding self-efficacy scale short form (BSES-SF), a 14-item questionnaire assessing a mother’s confidence in her ability to breastfeed her child [22]. Each item is a 5-point Likert scale from 1 (not confident at all) to 5 (very confident). Items are subsequently summed to obtain a total score, ranging from 14 to a maximum score of 70. Higher scores are indicative of higher breastfeeding self-efficacy. Since this data was not normally distributed, it was collapsed into a binary variable using the median score (56) to represent “high” or “low” breastfeeding self-efficacy.

### Confounding variables

Mothers were asked to report their age and date of birth and number of previous children, as well as their mode of delivery, infant’s date of birth and infant’s sex. Mothers were then asked about their socioeconomic status, including access to a series of 13 items as an indication of household assets. Mothers reported on their level of education attained, their employment status, and their relationship status. Mothers were also asked whether they had ever been diagnosed with human immune-deficiency virus (HIV), and if so whether they were taking antiretroviral medication.

Additionally, mothers completed the Health Literacy Questionnaire (HLQ) [23]. This questionnaire has 44 items across 9 scales that identify profiles of health literacy strengths and needs. For the purposes of this study, we extracted the data from 2 of the scales – ‘feeling understood and supported by healthcare providers’ (HPS) and ‘having sufficient information to manage health’ (HSI) – both are scored from 1 (strongly disagree) to 4 (strongly agree) and an average score for each scale is calculated. The HPS scale asks 4 questions related to having at least one trusted healthcare provider, where high scores indicate the participant has an established relationship with at least one healthcare provider who they trust and who provides useful information to help them make decisions about their health. People who score low on this scale are unable to engage with healthcare providers, and do not have regular healthcare providers, or have difficulty trusting providers and their sources of information or advice. The HSI scale asks 4 questions about having enough good information to manage health. Participants scoring high on this scale feel confident that they have the information they need to manage their health/conditions and make decisions, while participants who score low on this scale feel there are gaps in their knowledge and that they do not have the information they need to live with and manage their healthcare concerns. Both scales were considered high if the mean score was >= 2.5.

### Statistical analysis

**Fig. 1:**
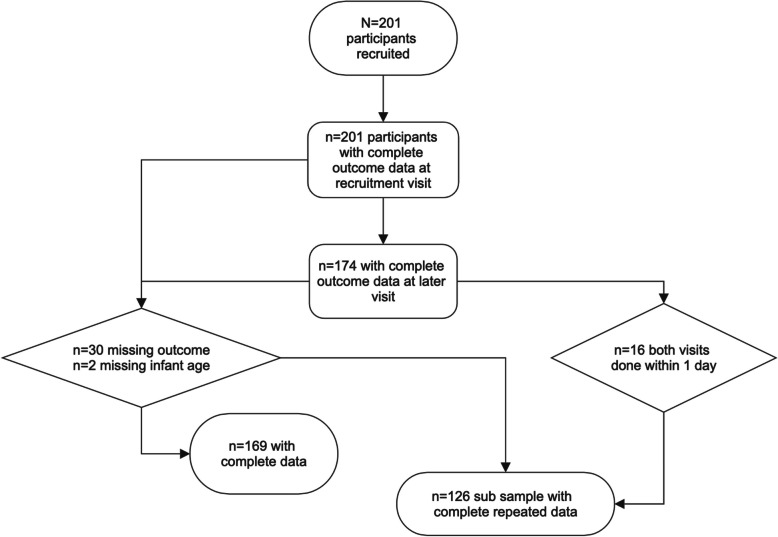
Consort diagram representing missing data and excluded participants

Participant characteristics and variables of interest were summarized and presented as mean(SD) or frequencies(percentage). Upon summarizing participant data, 32 participants had missing data and were excluded from the analysis leaving a sample size of (*n* = 169). For the subsample with repeated measures, if this second measurement was done on the same day as the first it was excluded, providing a subsample of (*n* = 126) with repeated data at a later timepoint (See Fig. 1).

In order to estimate the causal effect of mothers’ general perceptions of nurse support on exclusive breastfeeding, mediated via breastfeeding self-efficacy, G-computation for mediation analysis with Monte Carlo simulation was used. G-computation uses a counterfactual approach to mimic a randomized trial by simulating breastfeeding exclusivity (Y) as a function of all participants being exposed (X = 1; i.e.: finding nurses helpful ‘always’), and then as a function of all participants being unexposed (X = 0; i.e.: finding nurses helpful ‘sometimes, seldom or never’). Mediation effect decomposition then provides the total causal effect (TCE) which is a comparison between these means and mimics an average treatment effect. The natural direct effect (NDE) is then simulated similarly, but setting the mediator to its natural level if all participants were unexposed (M; X = 0), thus providing the effect via pathways other than through the mediator. The natural indirect effect then gives an estimate of the mediation effect and is calculated as TCE-NDE. Proportion mediated can be calculated as NIE/TCE [24]. These counterfactual approaches allow for estimation of the causal mediation effect using inference [25].

**Fig. 2:**
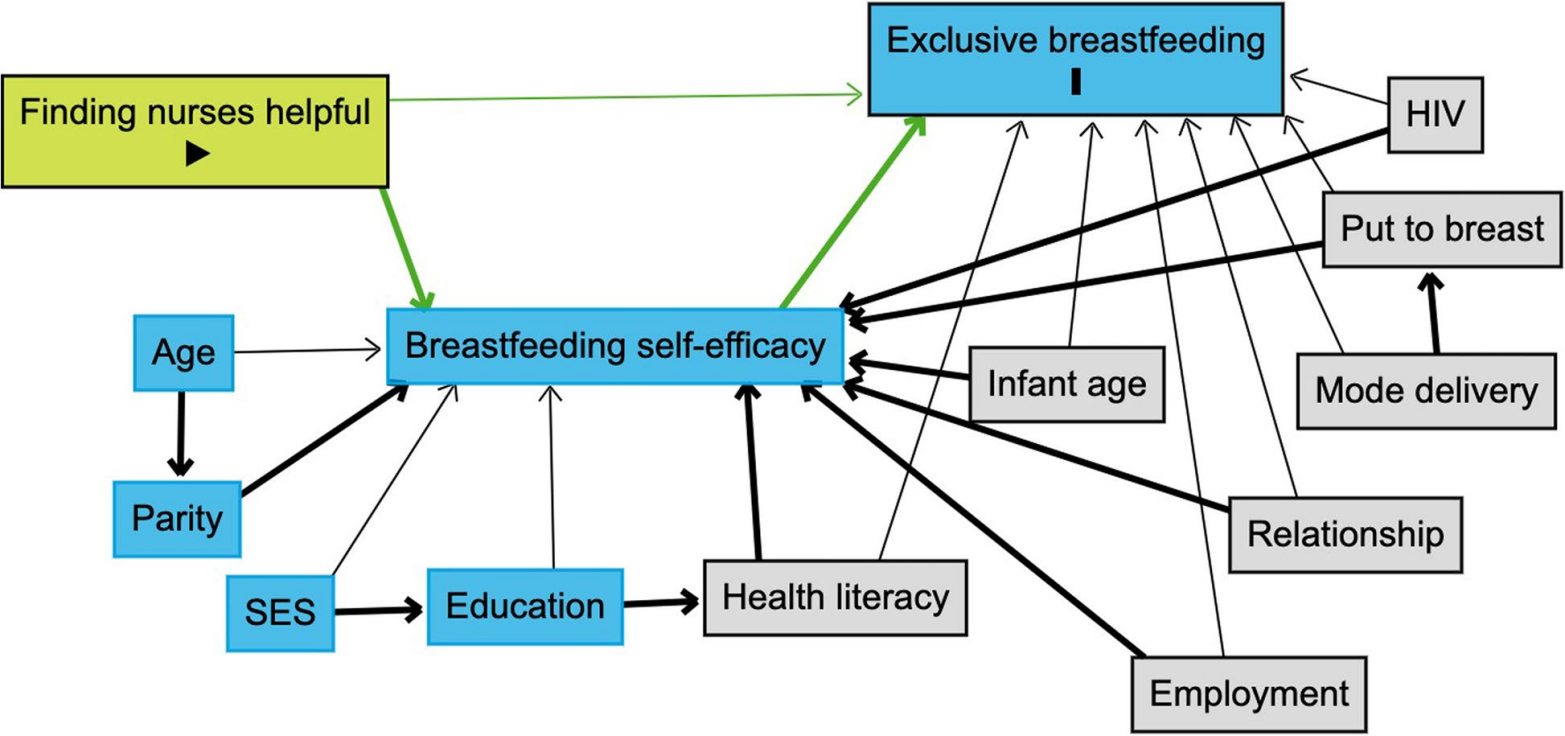
Directed acyclic graph showing a priori proposed mediation model. Green block represents exposure, blue blocks represent the mediator and outcome, and grey blocks represented confounders to be adjusted. Clear blocks represent variables involved in the conceptual model, which do not require adjustment based on rules of d-separation and minimal sufficient adjustment to test causal pathways. Green lines represent the causal paths. Drawn using Dagitty [26]

First, the directed acyclic graph (DAG) was drawn considering all a priori defined confounders and relationships (Fig. 2). Given the small sample size and to allow for model convergence, minimal sufficient adjustment was determined using the rules of d-separation and ensuring all paths besides the causal paths would be blocked by adjustment [26]. The final DAG to be tested is shown in Fig. 2 with variables to be adjusted indicated by the colour legend. Thereafter, each proposed pathway (Y←X, M←X, Y←C, and M←C; whereby X is the exposure “finding nurses helpful”, Y is the outcome “breastfeeding exclusivity”, and C represents the confounders of M or Y) were tested using logistics regressions.

The model was then tested. The M equations were specified as linear, and Y equations were specified as logistic. Next, a sensitivity analysis was conducted to consider relationships over a longer period of time in the sub sample with repeated data, thus accounting for presumably more decline in breastfeeding exclusivity. Here, the model was repeated using breastfeeding exclusivity, breastfeeding self-efficacy and infant age at the second visit. In all cases, 1000 bootstrapped samples were created to tighten the estimates, and bias corrected 95% confidence intervals were presented (along with normal confidence intervals). All analyses were done in Stata/SE V17.0 for Mac.

## Results

### Maternal and infant characteristics

Participant characteristics and breastfeeding practices are presented in Table 1. Maternal age ranged from 18–43 years old with a median of 29 years. At the time of assessment, infants had a median age of 4 days with a range of 1–46 days. While many women had completed high school, only 16% had completed further education. Less than a quarter of participants were employed, and 37% had never been employed. Most participants were single, or in a relationship but not married, and most mothers (66%) already had 2 or more children, with 11% reporting having 4 or more children. More than a quarter of the women delivered via caesarean (29%).

**Table 1:** Participant characteristics and breastfeeding practices

	Mean (SD)	n (%)
Maternal and infant variables		
Maternal Age (years)	29 (6)	
Infant age (days)	6 (6)	
Infant sex (female)		74 (44)
Mode of delivery (vaginal)		120 (71)
Household assets (score/13)	8 (2)	
Highest level of education		
Grade 9		1 (1)
Grade 10		11 (7)
Grade 11		49 (29)
Grade 12		108 (64)
Currently employed (yes)		42 (25)
Unemployed and currently looking for a job (yes)		52 (65)
Current relationship status		
Married		15 (9)
In a relationship living alone		51 (30)
Living with partner		43 (25)
Single		60 (36)
HIV (yes)		34 (20)
Healthcare support, health literacy and breastfeeding self-efficacy		
Breastfeeding self-efficacy score (/70)	64 (7)	
Nurses are helpful		
Always		72 (43)
Sometimes		85 (50)
Seldom		12 (7)
Health Literacy		
Feeling understood and supported by healthcare providers (high)		129 (76)
Having sufficient information to manage health (high)		119 (70)
Breastfeeding practices at first visit		
Baby put immediately to breast (yes)		124 (73)
Currently breastfeeding (yes)		150 (89)
Exclusively breastfeeding (yes)		132 (78)

### Healthcare support, health literacy and breastfeeding self-efficacy

The majority of participants (55%) scored 70/70 on the breastfeeding self-efficacy scale, and only 14% scored below 56/70 (lowest quartile). Less than half of the women reported the nurses to be helpful “Always”. Most participants ranked high on ‘Feeling understood and supported by healthcare providers’ and on ‘Having sufficient information to manage health’ from the health literacy questionnaire.

### Breastfeeding practices

Most data were collected within 4 days of birth (IQR: 3–7 days), with only 10% being collected after 12 days. While nearly 90% of women were breastfeeding at this time, only 78% were exclusively breastfeeding. Only 3% of the participants never initiated breastfeeding, and all those who stopped breastfeeding stopped within the first 3 days. Of those who stopped breastfeeding entirely (*n* = 19), reasons for stopping were often unspecified (47%). Those who specified their reasons stopped due to lactation problems or pain during breastfeeding (16%), medical complications (11%—labour complications, previous breast reduction surgery), and for pursuit of education/work (11%). While only 20% of women with HIV had stopped breastfeeding, 75% did so for fear of vertical transmission. Fifteen percent of infants were given formula, and in 80% of cases this happened within the first 3 days. Of those who were currently giving their infants formula 47% were mixed feeding (breastmilk and formula), but the reasons for supplementing with formula were not specified.

**Fig. 3:**
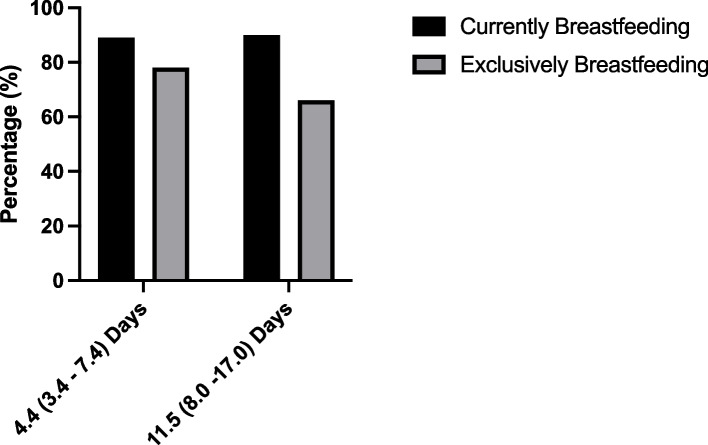
Breastfeeding practices at first and second (subsample) visit (median (IQR))

In the subsample who had data collected again at a later stage, in most cases (> 60%) this was done within 14 days, and in over 90% of cases was done within a month (data not shown). At this stage 90% of the participants were breastfeeding, yet only 66% were exclusively breastfeeding. All but one of the women who had discontinued breastfeeding or starting mixed feeding at this stage did so within 10 days of delivery. Lack of supply and cracked nipples were now provided as additional reasons for stopping, and at this stage 17% of participants were providing formula. Figure 3 shows the exclusive and current breastfeeding rates at the two visits.

### Mediation analysis

The results from the mediation analysis (Table 2) show that there was a total causal effect of finding nurses generally helpful on breastfeeding exclusivity, whereby finding nurses ‘always’ helpful would result in a 14% greater likelihood of exclusively breastfeeding (SE = 0.06, *p* = 0.03, BC 95% = 0.02 to 0.26). However, this effect was not mediated via breastfeeding self-efficacy (proportion mediated = 7%; NIE = 0.01, BC 95%: −0.00 to 0.05). In testing the model equations, it was clear that finding nurses helpful was not directly associated with breastfeeding self-efficacy (ß = −0.03, SE = 0.55, *p* = 0.96, 95%: −1.10 to 1.05), therefore it would be unlikely that any mediation effect would be seen. Instead, a direct effect via pathways other than breastfeeding self-efficacy was evident (*p* = 0.04, BC 95%: −0.00 to 0.24).

**Table 2:** G-computation estimates of causal effect (TCE) of finding nurse helpful on breastfeeding exclusivity and mediation effect via breastfeeding self-efficacy (NIE) in full sample (*n* = 169)

	G-estimates	Bootstrap SE	z-statistic	*p*-value	95% CI	95% CI (BC)
TCE	0.14	0.06	2.20	0.03	0.02 to 0.26	0.00 to 0.24
NDE	0.13	0.06	2.06	0.04	0.01 to 0.25	−0.01 to 0.24
NIE	0.01	0.01	0.55	0.58	−0.01 to 0.03	−0.00 to 0.05

Data was collected at 4(3–7) days post delivery

Total causal effect (TCE) is a comparison between counterfactual means and mimics an average treatment effect. Natural direct effect (NDE) provides the effect via pathways other than through the mediator. Natural indirect effect (NIE) gives an estimate of the mediation effect

In the sensitivity analysis (Table 3) the results were very similar with the total causal effect estimate becoming slightly stronger. In this model, the direct effect via pathways other than breastfeeding self-efficacy was not significant (*p* = 0.12, BC 95%: −0.03 to 0.31).

**Table 3:** G-computation estimates of causal effect (TCE) of finding nurses helpful on breastfeeding exclusivity and mediation effect via breastfeeding self-efficacy (NIE) in sub sample with repeated measures (*n* = 126)

	G-estimates	Bootstrap SE	z-statistic	p-value	95% CI	95% CI (BC)
TCE	0.18	0.08	2.16	0.03	0.02 to 0.34	0.02 to 0.34
NDE	0.14	0.09	1.57	0.12	−0.03 to 0.31	−0.04 to 0.30
NIE	0.04	0.03	1.4	0.16	−0.02 to 0.10	0.00 to 0.11

Data was collected at 11(8–17) days post delivery

Total causal effect (TCE) is a comparison between counterfactual means and mimics an average treatment effect. Natural direct effect (NDE) provides the effect via pathways other than through the mediator. Natural indirect effect (NIE) gives an estimate of the mediation effect

## Discussion

This study aimed to determine infant feeding practices shortly following delivery, and to estimate the causal effect of perceived general support from nurses on breastfeeding exclusivity, mediated via breastfeeding self-efficacy. The results showed that nearly a quarter of the mothers in this study were not exclusively breastfeeding within two weeks postpartum, and that cessation of breastfeeding or supplementation with formula usually happened within the first three days postpartum. Perceived general support from nurses was causally related to breastfeeding exclusivity whereby increased perceived support promoted exclusivity, however this relationship was not mediated via breastfeeding self-efficacy. These findings point to a very clear modifiable target for intervention, in that nurses should be trained to be approachable and helpful, and to better support and educate all mothers during the crucial first few days following delivery, regardless of their breastfeeding self-efficacy.

The exclusive breastfeeding rates found in this study are lower than in those reported in the first six weeks postpartum in Kenya and Zambia, as well as in other LMICs [27], but higher or similar to reported rates in South Africa in the first month [28, 29] (likely because this data was collected within only a few days with less time for cessation to occur). Most women in this study continued to breastfeed while supplementing with formula, thus the problem lies in exclusivity rather than uptake or continuation of breastfeeding. Reasons for stopping breastfeeding in almost all cases were due to modifiable factors which are common challenges or misconceptions affecting breastfeeding mothers around the world (perceived insufficient milk supply, pain, pursuit of work or education). Discontinuing breastfeeding due fears about HIV transmission points to a lack of information or misinformation about breastfeeding policies in South Africa for women living with HIV. This is understandable given the numerous and rapid policy changes that have occurred [30], resulting in hospital staff being influenced by old messaging, with some health facilities still displaying messaging aligned with previous policy that encourage formula use for women with HIV [9, 13]. Therefore, both mothers and hospital staff are confused by mixed messaging, which can result in fear and avoidance of breastfeeding.

The reasons for cessation of breastfeeding reported in this study are well documented in South Africa [14, 16, 28–33]. It is likely that formula was being given unnecessarily in most cases, given that only two mothers reported medical/labour complications, and that no mothers were being treated for TB, epilepsy, or diabetes. The process of breastfeeding, as well as breastmilk itself is highly specialised and adaptive ensuring optimized infant nutrition and healthy growth and development, which cannot be replicated artificially by breastmilk substitutes [1]. It is unknown why mixed feeding was happening in this study as mothers did not state reasons for giving formula, but misconceptions about perceived supply, infants’ hunger cues and nutritional requirements are often used in the promotion of formula milk [34] and likely have a role to play in the advice mothers are receiving about breastfeeding from family, friends and health workers. Such misconceptions about the need to supplement with formula are frequently reported by mothers in South Africa [14, 16, 28–31, 33]. These barriers to exclusive breastfeeding are therefore already known and should be anticipated and counteracted by healthcare workers. This highlights the need for better antenatal education to overcome common barriers that result in cessation of breastfeeding.

Nurses and healthcare workers are an important and prominent source of breastfeeding information for mothers [14, 28, 30, 31], and mothers who are provided with advice about breastfeeding are more likely to exclusively breastfeed [31]. Supportive nurses may promote breastfeeding self-efficacy, however perceived negative attitudes and abusive behaviour by nurses are frequently reported across a range of health care sectors, and specifically with regards to postpartum care and breastfeeding [14–16]. Indeed, in this study mothers perceiving the nurses to be generally helpful was causally related to breastfeeding exclusivity. However, counter to our hypothesis, this was not mediated by breastfeeding self-efficacy. Given that mothers reported their general perceptions of nurses’ helpfulness, this variable may not have specifically captured nurses’ attitudes towards breastfeeding support which may explain why breastfeeding self-efficacy was not associated. Regardless of the type and content of support nurses were offering/not offering, it was directly predictive of breastfeeding exclusivity. This may indicate that mothers who found nurses unhelpful were less likely to seek breastfeeding support, or that unhelpful nurses were less likely to be providing any post-partum support including breastfeeding guidance and education. In developing countries health workers are seen to be in a position of power, making their influence on mothers’ decision making powerful [13]. It may be that not receiving any postpartum support from nurses would make mothers more likely to seek and heed advice from unreliable sources, including advice about breastfeeding and mixed feeding. This would make them more susceptible to misinformation and inaccurate beliefs.

### Recommendations

Firstly, it is disturbing that so many mothers in South Africa report nurses’ attitudes as barriers to accessing proper healthcare and support. Clearly, some form of intervention is needed to educate nursing students and staff about patients’ rights and empathetic and quality care provision [15, 35, 36]. At the recent Johannesburg Health District Research Conference held in August 2024, research from all levels of healthcare consistently reported nurse attitudes and behaviours as significant barriers and deterrents regarding health seeking behaviours for mothers and patients in general [37]. Additionally, given the severely overburdened healthcare system in South Africa, and that healthcare workers often experience burnout and report limited support and ability to practice self-care [38–40], there is a clear need to also support nurses properly to allow them to effectively and compassionately do their work.

Secondly, ensuring that nursing staff have sufficient knowledge to support mothers in breastfeeding, and that mothers are being properly supported, educated, and counselled about breastfeeding following delivery is evidently critical to promote exclusivity. Nurses should be trained to support mothers with correct latching and continued lactation and educate them about their right to breastfeed regardless of school attendance or employment. Nursing staff should provide anticipatory guidance by dispelling common myths and addressing common concerns immediately post-partum, which would likely help mothers to overcome frequently reported challenges and promote exclusivity. A Cochrane review and meta-analysis on support for breastfeeding mothers highlighted that breastfeeding specific supportive interventions were most effective at preventing breastfeeding cessation in LMICs [41]. Breastfeeding specific supportive interventions had a beneficial effect on breastfeeding exclusivity at all months between 1 and 6 months, regardless of intensity of the intervention, who was delivering the intervention and mode of delivery [41], indicating that any form of intervention is beneficial. In the interventions included in the Cochrane review, almost all the intervention delivery personnel had received extra training in breastfeeding support [41]. It is recommended that nurses (and nurse students) in South Africa undertake a 20 h lactation course [9], which has been shown to have a great impact on attitudes towards BFHI [8]. Staff should also be trained on the code of marketing for breastmilk substitutes and should have clear recommendations regarding women with HIV. Some studies focusing specifically on HIV-positive women have shown that HIV-positive mothers have higher odds of maintaining breastfeeding exclusivity, possibly due to intensive mother-to-child transmission counselling received [30, 42]. Others have shown that exclusive breastfeeding is associated with the stigma of HIV, which can cause avoidance and mixed feeding practices [30]. Ultimately, it is imperative that all mothers are supported to breastfeed for as long as possible, and that HIV-positive mothers are counselled and supported to maintain viral suppression [43].

Thirdly, in the context of interventions to promote exclusivity for the first 6 months, it is important to note that most women had stopped breastfeeding entirely or supplemented with formula within three days. This is often prior to the initiation of any infant health promotion activities. In fact, opportunities for promoting exclusivity seem to be critical in the first three days. We therefore recommend that interventions to promote breastfeeding exclusivity need to happen prior to delivery, or immediately upon delivery. Furthermore, all clinics need to enforce the BFHI immediately to provide mothers with a chance at exclusivity. A study conducted in Limpopo examined nurses’ attitudes towards implementation of the BFHI in rural public healthcare facilities in South Africa and found that most enrolled auxiliary nurses showed negative beliefs indicating that they did not teach women about the benefits of breastfeeding, and were negative about the support, promotion and protection of breastfeeding [8]. There was also indication that nurses had incorrect knowledge about breastfeeding exclusivity and introduction of solids [8]. Monitoring of implementation of the BFHI is thus required, and regular retraining is recommended. These recommendations are not novel, but evidently need to be restated.

This study did have some limitations. Although the question used to assess perceived helpfulness of nurses was general and did not specifically ask about *breastfeeding* support, this study aimed to assess whether general perceptions of nurse support were influential on breastfeeding practices, given the frequent reporting of poor support from nurses for maternal care. Furthermore, it was administered within days of birth during which time mothers would be requiring assistance from clinic sisters with breastfeeding, as well as postnatal care. Also, the exclusivity rates reported are at the point of data collection, which was not consistent between participants, yet there was little variability in how long post-delivery data were collected, and this was adjusted for in the analysis. It was unexpected that the relationship between perceived support and breastfeeding exclusivity was not mediated via breastfeeding self-efficacy, however breastfeeding self-efficacy rates were exceptionally high in this sample and this lack of variability as well as a small sample size should be considered in interpretation. However, attempts were made to ensure model validity and good fit such as only controlling for minimally sufficient confounders, increasing simulations to reduce Monte Carlo error, and reporting bias corrected confidence intervals.

## Conclusions

We have shown that almost a quarter of women discontinued breastfeeding or supplemented with formula within the first few days following delivery. It is therefore impossible for these women to meet the guidelines of exclusive breastfeeding for at least 6 months, even though their breastfeeding self-efficacy was very high. Importantly, perceived helpfulness of nurses had a direct impact on mothers’ breastfeeding decisions. Nurses should be trained to provide support, knowledge, and anticipatory guidance for mothers in order to dispel myths, help with common challenges, and ultimately improve exclusivity rates. Additionally, nurses need to be better supported to manage their attitudes and behaviours towards patients. Promotion of breastfeeding exclusivity needs to happen immediately upon delivery, or even sooner; and monitoring and enforcement of the BFHI must be done regularly. The National Department of Health in South Africa needs to take charge and prioritise breastfeeding promotion and support in order to protect the health and wellbeing of mothers and children.

## Supplementary Information

Supplementary Material 1.

## Data Availability

The datasets used and/or analysed during the current study are available from the corresponding author.

## Abbreviations

BFHI: Baby-Friendly Hospital Initiative
BSES-SF: Breastfeeding self-efficacy scale short form
DAG: Directed acyclic graph
HIV: Human immune-deficiency virus
HLQ: Health literacy questionnaire
HPS: Feeling understood and supported by healthcare providers
HIS: Having sufficient information to manage health
LMIC: Low-to-middle-income
NDI: Natural direct effect
NIE: Natural indirect effect
PLAY: Play, Love, And You trial
TB: Tuberculosis
TCE: Total causal effect
