# Supplementary material for: An estimation of the effect of finding nurses helpful on early breastfeeding exclusivity mediated via breastfeeding self-efficacy, using a counterfactual approach and G-computation
Source: BMC Pregnancy Childbirth. 2025 Dec 30;25:1354. doi: 10.1186/s12884-025-08429-8 (PMC12750716; doi:10.1186/s12884-025-08429-8)
Supplement: Supplementary file 1 — Supplementary Material 1. [file 12884_2025_8429_MOESM1_ESM.pdf]

Infant Feeding Practices (hospital)

Record ID ______________________________

______

Breastfeeding Questions (except from the WHO IYCF Questionnaire)

____

1.1 Was your baby put to your breast within the first hour of giving birth?

(Go to 1.2)

◯ Yes
◯ No

1. 2 Are you currently Breastfeeding your baby?

(Yes: Go to 3, No: Go to 2)

◯ Yes
◯ No

2. At what age did you stop breastfeeding your baby?

______ Days
______ Weeks
______ Months
(Go to 4)

Days + Weeks*7 + Months*30,417 ______________________________

3. Have you ever giving your baby anything other than breastmilk?

(Yes: Go to 5, No: Go to 9)

◯ Yes
◯ No

4. What were your reasons for stopping to breastfeed / not breastfeed your baby?

(Go to 5)

☐ Work
☐ Education
☐ Illness
☐ Lactation problems
☐ Child not growing well
☐ Child crying a lot
☐ Not enough breast milk
☐ Did not want to give my baby HIV infection
☐ Advice/pressure from others
☐ Other

Specify ______________________________

5.1 Have you ever Formula Fed your baby?

(Yes: Go to 5.2, No: Go to 6)

◯ Yes
◯ No

5.2 Are you currently Formula Feeding your baby?

(Yes: Go to 6, No: Go to 5.3)

◯ Yes
◯ No

5.3 How old was the Baby when you stopped giving him / her formula milk?

Days ______

Weeks ______

Months ______

(Go to 6)

Days + Weeks*7 + Months*30.417 ________________________________

6. How old was the baby when you FIRST gave him / her anything other than breast milk or medicine. In other words, how old when he/she first had any water or food or formula milk

Days ______

Weeks ______

Months ______

(Go to 7)

Days + Weeks*7 + Months*30.417 ________________________________

7. What was the first food/drink, other than Breastmilk that you gave your baby? ________________________________

(Go to 8)

8. From the time you woke up yesterday morning, to the time you woke up this morning, did you give any of the following items to the child?

If so, how often did you give each of these items (from the time you woke up yesterday morning to the time you woke up this morning)?

(Go to 9)

- ☐ Water
- ☐ Water with sugar
- ☐ Fruit juice
- ☐ Herbs in water
- ☐ Tea without milk
- ☐ Tea with milk
- ☐ Diluted cows milk
- ☐ Non-diluted cows milk
- ☐ Infant formula
- ☐ Other powdered milk
- ☐ Dairy products like yoghurt, cheese, cream
- ☐ Goats milk
- ☐ Cereals, porridge or bread
- ☐ Any fruits / vegetables
- ☐ Any meat or fish
- ☐ Eggs
- ☐ Gripe Water
- ☐ Any prescribed medicine
- ☐ Any non-prescribed medicine
- ☐ Any alcohol like beer or brew
- ☐ None of the above
- ☐ Other

How often water ________________________________

How often sugarwater ________________________________

How often fruit juice ______________________

How often Herbs in water ______________________

How often tea no milk ______________________

How often tea+milk ______________________

How often diluted cows milk ______________________

How often non-diluted cows milk ______________________

How often infant formula ______________________

How often other powdered milk ______________________

How often dairy ______________________

How often goats milk ______________________

How often porridge/bread ______________________

How often fruit/veg ______________________

How often meat/fish ______________________

How often eggs ______________________

How often gripe water ______________________

How often prescribed meds ______________________

How often non-prescribed meds ______________________

How often alcohol ______________________

Other, specify	__________________________________
How often other	__________________________________
9. Which of these medicines is your baby currently receiving:<br><br>Tick all that apply	☐ Multivitamins<br>☐ Iron Drops<br>☐ Zinc Syrup<br>☐ Nevirapine<br>☐ Co-Trimoxazole (or Bactrim / Trimethoprim-Sulphamethoxazole / Resmed / Iantibiotic)<br>☐ TB Drugs<br>☐ Antibiotics<br>☐ Other - specify<br>☐ Baby is not currently receiving any medicine
Other_medication_specify	__________________________________
